# Dr. John R. Selover

**Published:** 1913-04

**Authors:** 


Dr. John R. Selover was born at Enfield, Tompkins Co., N. Y., Jan. 8, 1824. His family moved to Ohio, in wagons, while he was a boy, but returned after a few years to Trumansburg, N. Y., where he acquired a practical knowledge of tinsmithing, after which he engaged in the hardware business in Prattsburg. He spent two years in California during the gold fever, returning to Prattsburg and studying dentistry with Dr. P. K. Stoddard. In 1874 he graduated in medicine from the University of Buffalo and, after an experience as surgeon to a Mexican mine, he finally settled in Trumansburg, continuing in the practice of medicine and dentistry till his death, Feb. 26, 1913, in his ninetieth year. He was well versed in astronomy and geology, and had acquired a fluent use of Spanish. He was a man of great native ability and highly esteemed by his associates and patients. His daughter, Marion, married the late Dr. E. H. Tweedy of Buffalo.

